# Combinational Therapy Enhances the Effects of Anti-IGF-1R mAb Figitumumab to Target Small Cell Lung Cancer

**DOI:** 10.1371/journal.pone.0135844

**Published:** 2015-08-19

**Authors:** Hongxin Cao, Wei Dong, Hongchang Shen, Jun Xu, Linhai Zhu, Qi Liu, Jiajun Du

**Affiliations:** 1 Institute of Oncology, Shandong Provincial Hospital Affiliated to Shandong University, Shandong University, Jinan, P.R. China; 2 Department of Thoracic Surgery, Shandong Provincial Hospital Affiliated to Shandong University, Shandong University, Jinan, P.R. China; Thomas Jefferson University, UNITED STATES

## Abstract

**Background:**

Small cell lung cancer (SCLC) is a recalcitrant malignancy with distinct biologic properties. Antibody targeting therapy has been actively investigated as a new drug modality.

**Methods:**

We tested the expression of IGF-1R and calculated the survival in 61 SCLC patients. We also evaluated the anti-tumor effects of anti-IGF-1R monoclonal antibody Figitumumab (CP) on SCLC, and tried two drug combinations to improve CP therapy.

**Results:**

Our clinical data suggested that high IGF-1R expression was correlated with low SCLC patient survival. We then demonstrated the effect of CP was likely through IGF-1R blockage and down-regulation without IGF-1R auto-phosphorylation and PI3K/AKT activation. However, we observed elevated MEK/ERK activation upon CP treatment in SCLC cells, and this MEK/ERK activation was enhanced by ß-arrestin1 knockdown while attenuated by ß-arrestin2 knockdown. We found both MEK/ERK inhibitor and metformin could enhance CP treatment in SCLC cells. We further illustrated the additive effect of metformin was likely through promoting further IGF-1R down-regulation.

**Conclusion:**

Our results highlighted the potential of anti-IGF-1R therapy and the adjuvant therapy strategy with either MEK/ERK inhibitor or metformin to target SCLC, warranting further studies.

## Introduction

Small cell lung cancer (SCLC) is a recalcitrant malignancy with high recurrence and low five-year survival rate under conventional chemotherapy and radiotherapy [1]. Treatment of SCLC is challenging due to its rapid growth rate and the development of drug resistance during the course of the disease. SCLC possesses some distinct molecular and cellular changes that lead to its pathogenesis, including mutations/deletions of some tumor suppressors, activation of several oncogenes, abnormal activities of some developmental pathways, and up-regulation of certain receptor tyrosine kinases (RTKs) [2]. Because of the unique pathological/biological features of SCLC, seeking for new-targeted therapy is of high priority. As a rapidly expanding drug modality, antibody drugs against RTKs have been actively investigated for the treatment of SCLC, and insulin-like growth factor receptor (IGF-1R) is one of such potential RTK targets [3–7].

IGF-1R and its ligands are usually expressed at increased levels in SCLC, and are reported to correlate with poor prognosis [8,9]. There is preliminary evidence that the IGF-1R signaling pathway plays crucial roles in mitogenesis, anti-apoptosis, malignant transformation and metastatic events [10,11]. IGF-1R is now considered to be an attractive target for cancer treatment and there are some ongoing clinical trials testing the IGF-1R-targeted drugs. Figitumumab (CP-751, 871, CP), a human anti-IGF-IR monoclonal antibody (mAb), is proved to have anti-proliferation and anti-tumorigenicity effects in cancer cells and xenografted mice, and it has been showed to be effective in combination with other cytotoxic agents to target many cancer types [12–14]. CP was investigated in a Phase II clinical trial in combination with etoposide and cisplatin as a first-line treatment for extensive stage SCLC (NCT00977561). However this trial was prematurely terminated on 2011 due to slow enrollment of patients. To encourage the resume of clinical trial, the molecular mechanism of how CP targets SCLC is necessary. In addition, combining CP with other drugs to increase its efficacy is also critical to convince patients to enroll into the clinical trials.

Metformin, a widely used anti-diabetic drug derived from French lilac, has caloric restriction action on cell metabolism. Recently metformin is emerging as a candidate anti-cancer agent. Accumulation evidence has suggested that metformin has anti-cancer effects in leukemic, head and neck squamous cell carcinoma, prostate cancer, breast cancer, lung cancer and other solid tumors, although its precise mechanisms remain unresolved [15–20].

Malignant cells usually have higher glucose uptake rate and increased glycolysis to fulfill their metabolic requirement of rapid protein synthesis and cell proliferation. Unfortunately, hyperglycemia is reported to be one of the most highly occurred adverse events in clinical trials of anti-IGF-1R mAb therapy, which might benefit tumor cell growth and lower the efficacy the drug [21]. Because metformin has both hypo-glycemic and anti-cancer effects, it becomes a promising candidate in combination with anti-IGF-1R mAbs to target SCLC.

In addition to the potential usage of metformin, another group demonstrated that inhibition of the MEK/ERK signaling pathways promoted the effects of CP on esophageal carcinoma [22]. MEK/ERK inhibitors have been used alone or combined with other drugs to treat multiple cancers, such as sensitizing radiotherapy and/or enhancing chemotherapy. Combining Selumetinib (AZD6244), a MEK1/2 inhibitor, with conventional chemotherapeutic agents enhanced their efficacy to target different tumor xenografts [23]. In a NSCLC model, the use of Selumetinib resulted in decreased VEGF expression/activation, and coupling MEK and VEGFR inhibitors further inhibited tumor angiogenesis, growth, and metastasis [24]. In addition, combining OSI-906 (an IGF-1R/insulin inhibitor) with MEK 1/2 inhibitors (U0126 and selumetinib) showed synergistic anti-proliferative effects to target colorectal cancer cells [25]. Given their success in multiple cancers, it is worth to investigate whether MEK/ERK inhibitors could improve CP-based therapy in SCLC.

Herein, we investigated the molecular mechanisms of the antitumor effects of CP in SCLC and demonstrated that combining CP with either MEK/ERK inhibitor U0126 or metformin could enhance the therapeutic effects of CP to target SCLC.

## Materials and Methods

### Patients and specimens

The present study was conducted retrospectively on consecutive patients with primary SCLC who had undergone a surgical resection between January 2007 and December 2010 in Shandong Provincial Hospital. This study was reviewed and approved by the Ethical Committee of Shandong Provincial Hospital, and written informed consent was given by participants. Eligibility criteria included histological diagnosis of SCLC, surgical resection of the primary lesion and no chemotherapy or radiotherapy prior to surgery. Patients who died within one month after surgery, or with positive surgical margin were excluded. Finally, 61 archival formalin-fixed paraffin-embedded tumor samples were obtained.

Staging was determined based on the 7th edition tumor–node–metastasis (TNM) classification for lung cancer [26]. The following information including age, gender, smoking status, tumor location, tumor size, pathologic, and TNM stage was collected. All patients were treated according to National Comprehensive Cancer Network (NCCN) guidelines. Patients were followed up every 3 months within 1 year after surgery, every 6 months for 3 years, and every year thereafter.

The first end point was overall survival (OS), defined as the time from operation to the date of death, the date lost to follow-up or the date of latest follow-up. The second end point was disease-specific survival (DSS), and failure was defined as death from lung cancer or treatment-related complications. Additional end point included disease-free survival (DFS), defined as the time interval between the date of the definitive resection and detection of first disease recurrence, metastasis, or the date of the last follow-up.

### Immunohistochemical (IHC) Analysis of tumor specimens

Immunohistochemistry (IHC) for IGF-1R and Ki-67 was performed. Details of IHC analysis have been described previously [27]. IGF-1R expression was scored by determining the intensity and percentage of positive cells. Intensity was scored as negative (0), weak (+1), moderate (+2), or strong (+3). The extension of positive cells was scored into six categories: no staining (0), 1–5 (+1), 5–25 (+2), 25–50 (+3), 50–75 (+4), and 75–100% (+5) positive cells. An immunoreactivity score (IRS) was calculated by multiplying the staining intensity and the extension, resulting in a scale from 0 to 15. The IRS was divided into two groups: negative or low staining (IRS 0–5) and positive or high staining (IRS 6–15). Ki-67 staining was scored as the percentage of positive nuclear staining cells and the cutoff level subdivided according to the percentages of nuclear staining as 50% positive.

### Reagents

IGF-1Rß, phospho-IGF-1R (Tyr1135/1136), phosphor-p44/42 MAPK (Thr202/Tyr204) and phospho-Akt (Ser473) mAbs were purchased from Cell Signaling Technology. Anti-ß-arrestin1 and anti-ß-arrestin2 mAbs were from Abcam. Anti-GAPDH mAb was from Santa Cruz. Human IGF-1 and metformin were purchased from Sigma. Figitumumab was provided by Pfizer.

### Cell Culture

H446, H526 and SKBR3 cells were purchased from ATCC. H446 and H526 were maintained in RPMI-H1640 medium supplemented with 10% Fetal bovine serum (FBS). SKBR3 was maintained in DMEM supplemented with 10% FBS.

### Transfection

The siRNAs against ß-arrestin-1 and ß-arrestin-2 were obtained from GenePharma (Shanghai, China). Transient transfection of siRNAs (30nM) was performed using Lipofectamine RNAiMAX (Invitrogen) according to the manufacturer’s protocol.

### Western Blotting Assay

Protein samples were dissolved in lithium dodecyl sulfate sample buffer (Invitrogen) and equal amounts of samples were separated by SDS/PAGE. Details of Western blotting analysis have been described previously[22].

### Densitometry Analysis

Band intensity was quantified by ImageJ and the results were normalized to the corresponding loading controls.

### Cell Viability Assay

Cell proliferation was measured with MTT (Sigma Chemical). Cells were incubated in 96-well plates, and after appropriate treatment time, cell viability was tested according to manufacturer’s protocol. Triplicates were performed for each treatment.

### Statistical Analysis

Differences in patient characteristics per group and co-expression of markers were calculated by Fisher’s exact test. Survival curves were calculated according to the Kaplan–Meier method and compared by the log-rank test. To identify prognostic factors for OS and DFS, univariate Cox regression analyses were performed. Student t test or ANOVA was performed to correct for multiple comparisons, as appropriated. All statistical tests were two-sided and were done using SPSS software 19.0 (SPSS Inc., Chicago, IL).

## Results

### IGF-1R expression level negatively correlates with survival of SCLC patients

**Fig 1:**
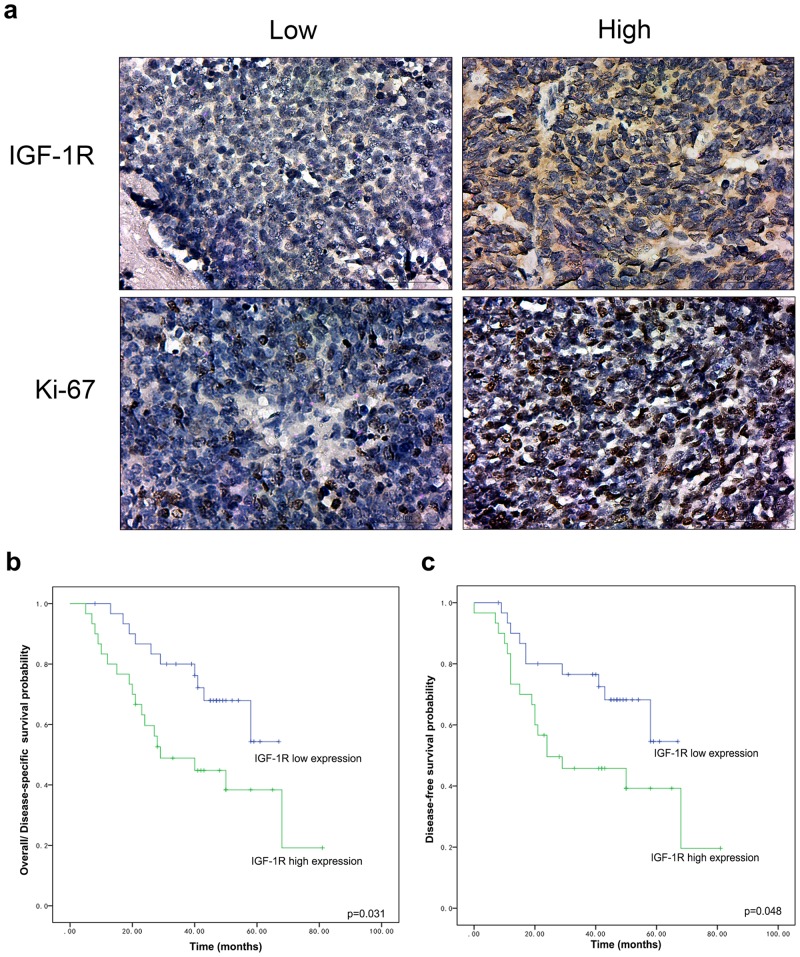
Representative immunohistochemical staining and survival curve of SCLC patients. **(a)** Immunohistochemical staining of IGF-1R and Ki-67 (x400) in SCLC patient samples according to the expression level of IGF-1R. **(b)** Overall survival/disease-specific survival in SCLC according to low- and high-IGF-1R expression. **(c)** Disease-free survival in SCLC according to low- and high-IGF-1R expression.

We first investigated whether IGF-1R expression level could predict the survival of SCLC patients. A total of 61 patients were included in our study, including 39 men and 22 women, with a mean age of 57.21 years (range 30–75 years). The median follow-up period was 40 months, ranging from 5 to 81 months. The main clinical characteristics of the patients were listed in Table 1 and the representative immunohistochemical staining was showed in Fig 1a. In the correlative analysis between IGF-1R expression and clinical parameters, IGF-1R expression was significantly associated with tumor size, N status, stage, and Ki-67 (Table 1).

**Table 1:** Characteristics of patients and correlation between IGF-1R expression and clinic-pathological characters.

		IGF-1R		
Characteristics	No. of patients (%)	Low	High	p-value
Sex				0.791
Male	39	19	20	
Female	22	12	10	
Age				0.384
≤65	46	25	21	
>65	15	6	9	
Size				0.041
<5 cm	31	20	11	
≥5 cm	30	11	19	
N status				0.026
N0	19	14	5	
N+	42	17	25	
Stage				0.214
I	13	7	6	
II	22	14	8	
III	26	10	16	
Ki67				0.021
Low	31	20	11	
High	30	10	20	
Smoking status				0.184
Smokers	22	14	8	
Nonsmokers	39	17	22	
Alcohol				0.426
Alcohol	21	9	12	
Nonalcohol	40	22	18	

We evaluated the prognostic value of IGF-1R expression related to three end points: overall survival (OS), disease-specific survival (DSS), and disease-free survival (DFS). We found that all deaths were SCLC-related, so DSS had the same result as OS. Kaplan–Meier curves of the two patient groups for OS/DSS were shown in Fig 1b. OS/DSS was significantly lower in patients with IGF-1R-low tumors than those with IGF-1R-high tumors (P = 0.031). Similar results were also obtained for DFS analysis (p = 0.048) (Fig 1c). Our results demonstrated the potential use of IGF-1R as a marker of poor prognosis for SCLC. Furthermore, we used Cox proportional hazards regression models to determine the independent predictors for OS/DSS and DFS (Table 2). Sex, stage, and IGF-1R expression were significant for OS/DSS (p = 0.006, 0.039, 0.037) in the univariate Cox analysis, while only IGF-1R expression was found to be an independent predictor in the multivariate Cox analysis (p = 0.037). For DFS, sex, stage, and IGF-1R expression was statistically significant in univariate Cox analysis (p = 0.002, 0.023, 0.048), while no parameters was significant in the multivariate Cox analysis. Overall, our clinical data suggested that IGF-1R expression level negatively correlated with survival of patients, implying that IGF-1R-targeting therapy, such as CP, has potential therapeutic values.

**Table 2:** Cox proportional hazard analysis.

	Univariate		Multivariate	
	HR (95% CI)	*P*-value	HR (95% CI)	*P*-value
**Overall survival**				
Sex		0.006		a
Male	1			
Female	0.187(0.056–0.621)			
Age		0.715		
≤65	1			
>65	0.830(0.306–2.256)			
Size		0.381		
<5 cm	1			
≥5 cm	1.538(0.587–4.029)			
N status		0.246		
N0	1			
N+	2.090(0.601–7.266)			
Stage		0.039		a
I	1			
II	4.120(0.918–18.500)			
III	4.533(1.081–19.000)			
Ki67		0.656		
Low	1			
High	1.234 (0.490–3.105)			
Smoking status		0.428		
Nonsmokers	1			
Smokers	1.599(0.500–5.114)			
Alcohol		0.265		
Nonalcohol	1			
Alcohol	1.707(0.667–4.369)			
IGF-1R		0.037		0.037
Low	1		1	
High	2.739(1.064–7.050)		2.307(1.053–5.052)	
**Disease-free survival**				
Sex		0.002		a
Male	1			
Female	0.152(0.045–0.512)			
Age		0.367		
≤65	1			
>65	0.641(0.244–1.685)			
Size		0.603		
<5 cm	1			
≥5 cm	1.295(0.490–3.425)			
N status		0.382		
N0	1			
N+	1.720(0.510–5.800)			
TNM		0.023		a
I	1			
II	3.963(0.910–17.264)			
III	5.375(1.261–22.912)			
Ki67		0.924		
Low	1			
High	1.045 (0.423–2.585)			
Smoking status		0.557		
Nonsmokers	1			
Smokers	1.422(0.439–4.606)			
Alcohol		0.174		
Nonalcohol	1			
Alcohol	1.913 (0.751–4.868)			
IGF-1R		0.048		a
Low	1			
High	2.611(1.007–6.774)			

^**a**^ Not in the final step of multivariate analysis

### Antitumor effects of CP in SCLC cell lines

**Fig 2:**
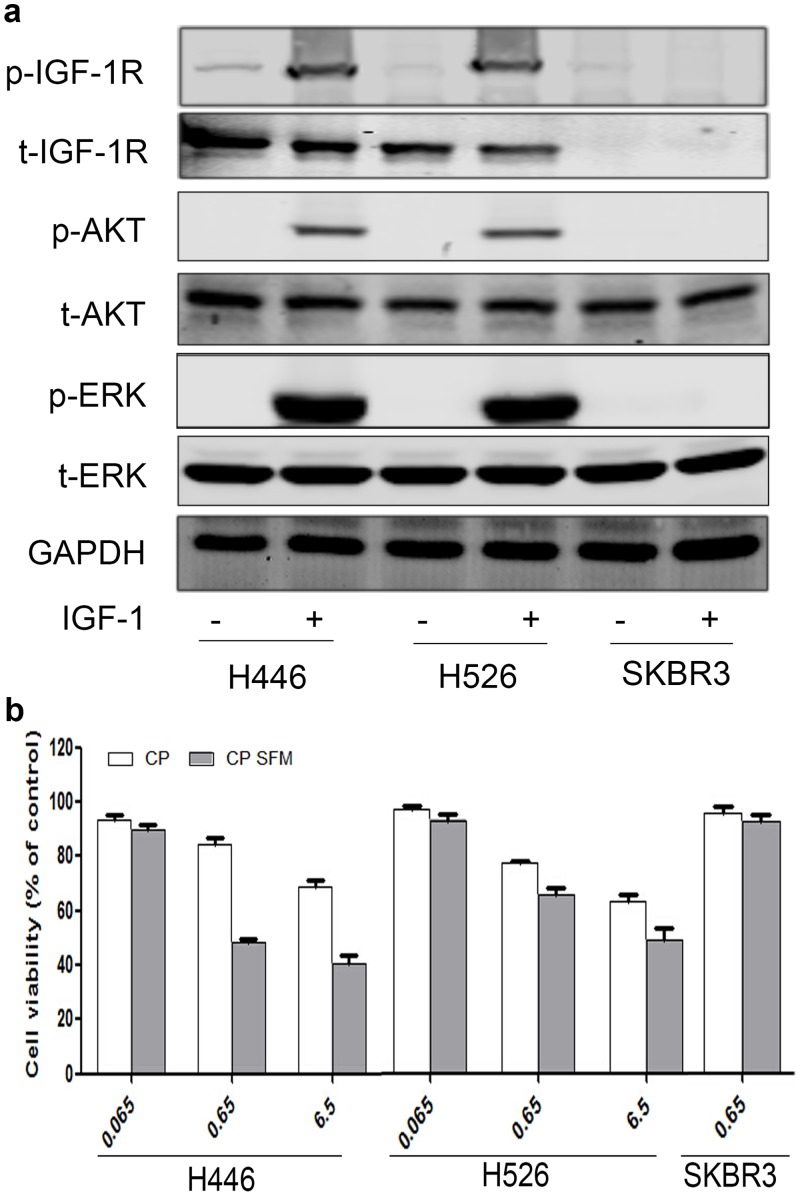
Sensitivity to CP treatment in SCLC cell line. **(a)** Cells were starved for 12h and stimulated with 6.5nM IGF-1 for 10min. p-IGF-1R, t-IGF-1R, p-ERK, t-ERK, p-AKT, t-AKT and GAPDH were detected by WB. **(b)** Cells were treated with 0.065nM, 0.65nM, and 6.5nM of CP for 48h with presence or absence of serum. Cell viability was tested through MTT. The number of viable cells following CP treatment was presented as percentage of untreated cells. We did each experiment for three times, and data were represented as mean±SEM. Statistical analysis: *P<0.05, **P<0.01, ***P<0.001.

Since IGF-1R is likely to play an important role in SCLC, inhibition of IGF-1R by CP, which competes with IGF to bind the receptor, might be useful to treat SCLC. We chose H446 and h526, typical SCLC cell lines, to investigate the effects of CP. As shown in Fig 2a,IGF-1 stimulation resulted in IGF-1R auto-phosphorylation and activation of downstream PI3K/AKT and MEK/ERK signaling pathways in H446 and H526 cells, while SKBR3, a breast cancer cell line known to express low amount of IGF-1R, did not response well.

We then investigated the sensitivity of SCLC cell lines to CP treatment. As shown in Fig 2b, after treated with 0.65nM CP, H446 and H526 cell viability compared with untreated control cells were 84.2% vs. 48.6% and 75.1% vs. 63.9% in the serum-added medium and serum-free medium (SFM), respectively, whereas SKBR3 cell viability didn’t change much upon CP treatment. This implies that the effects of CP are IGF-1R dependent. The SFM group was supposed to shield the competition of IGF-1 contained in normal serum medium. CP was originally designed to compete with IGF1 for IGF-1R to prevent receptor activation, and the anti-proliferative effect in serum-added medium supported this theory. However, the anti-proliferative effect in SFM conditions suggested that CP could either further suppress some basal IGF-1R activity that was undetectable by Western blot, or it might have other anti-proliferation/apoptosis effects independent of IGF-1R auto-phosphorylation/activation.

### CP induced IGF-1R endocytosis/down-regulation with increased ERK activation but not for IGF-1R and PI3K/AKT

**Fig 3:**
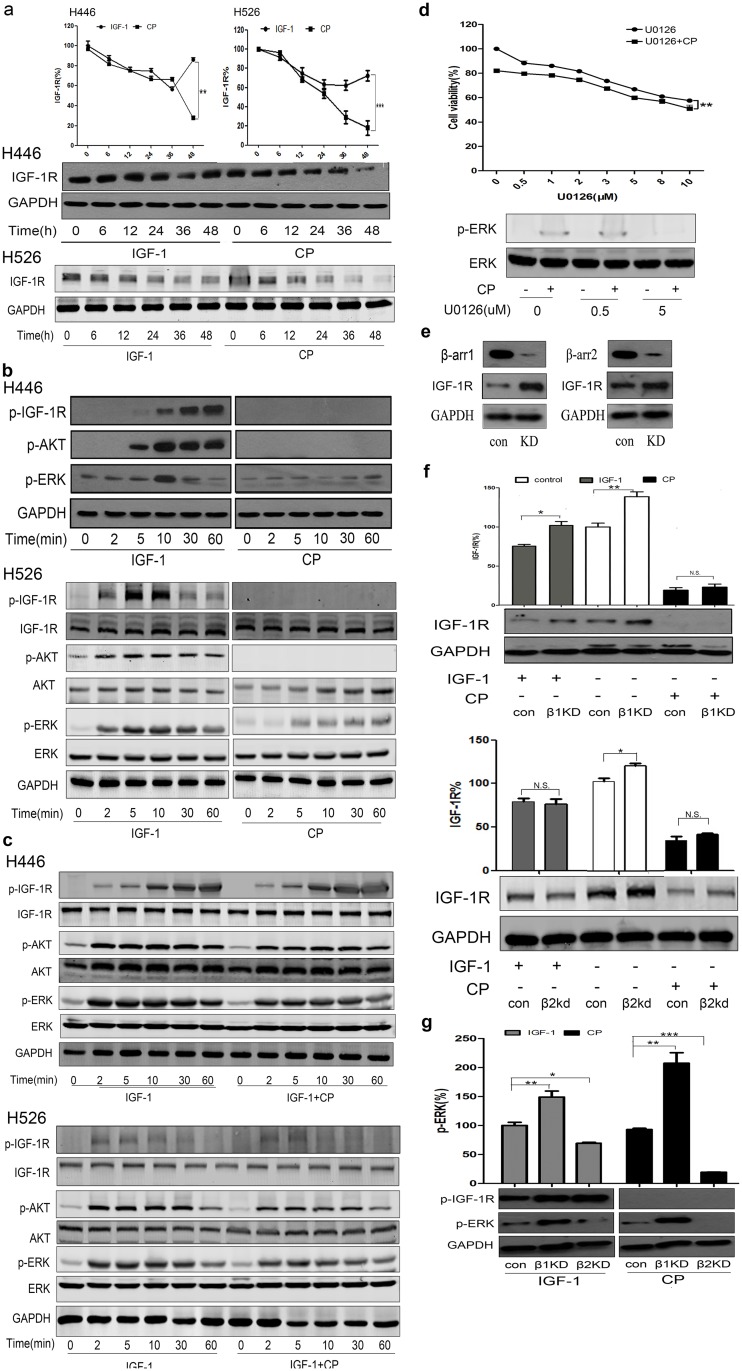
Inhibition of ERK activation enhances the therapeutic effect of CP. **(a)** H446 and H526 cells were serum starved for 12h and treated with either 0.65nM CP or 6.5nM IGF-1. IGF-1R and GAPDH were detected via WB. Intensity of IGF-1R were quantified by densitometry, normalized to GAPDH, and displayed as a percentage of the intensity at 0h. **(b)** Cells were starved for 12h and treated with 6.5nM IGF-1 or 0.65nM CP for a series of time points. Cell lysates were analyzed via WB for p-IGF-1R,t-IGF-1R, p-AKT,t-IGF-1R, p-ERK, t-ERK and GAPDH. **(c)** Cells were starved for 12h and treated with 6.5nM IGF-1 or 6.5nM IGF-1 plus 0.65nM CP for a series of time points. Cell lysates were analyzed via WB for p-IGF-1R,t-IGF-1R, p-AKT,t-IGF-1R, p-ERK, t-ERK and GAPDH. **(d)** Cells were treated with U0126 (0, 0.5, 1, 2, 3, 5, 8, 10 μM) for 60min, and then incubated with or without 0.65nM CP for 48h. Cell viability was tested via MTT. Cell lysates were prepared with different concentrations of U0126 with or without CP, p-ERK and t-ERK were analyzed by WB. **(e)** Knock down of ß-arr1 and ß-arr2 by siRNA in H446 cells. **(f)** H446 cells were knocked down for either ß-arr1 or ß-arr2. Cells were then starved for 12h, treated with 0.65nM CP or 6.5nM IGF-1 for 24h. Cells transfected with scrambled siRNA as control. IGF-1R and GAPDH were detected via WB. **(g)** H446 cells were knocked down for either ß-arr1 or ß-arr2. Cells were then starved for 12h, treated with 0.65nM CP or 6.5nM IGF-1 for 10min. Cells transfected with scrambled siRNA as control. p-IGF-1R, p-ERK and GAPDH were detected via WB. We did each experiment for three times, and data were represented as mean±SEM. Statistical analysis: *P<0.05, **P<0.01, ***P<0.001.

As some mAbs could induce the targeting receptor endocytosis and down-regulation, we investigated if CP has similar effects. As demonstrated in Fig 3a, CP could down-regulate IGF-1R in a time-dependent manner, which is similar to IGF-1-induced receptor endocytosis/ degradation. Interestingly, compared with IGF-1, CP continuously decreased IGF-1R expression while IGF-1R started to express back after 48hrs of IGF-1 stimulation (Fig 3a), suggesting a potent and sustained effect of CP on IGF-1R down-regulation.

Growth factor-induced receptor endocytosis is critical for RTK signal transduction and inhibition of endocytosis could attenuate downstream signaling pathways [28]. Because CP seems to have a similar effect as IGF-1 on IGF-1R endocytosis and degradation, we investigated if CP treatment could alter the downstream signaling pathways. As shown in Fig 3b, after cell starvation, IGF-1 could activate IGF-1R signaling pathway in a time-dependent manner, with pAKT and pERK peaked after 10 min of stimulation. However, CP failed to induce the auto-phosphorylation of IGF-1R or activate PI3K/AKT (Fig 3b). Activation of ERK was slightly higher, especially after 60min of CP treatment, but it was still much weaker than that activated by IGF-1 (Fig 3b). Further, we investigated the effect of CP on IGF-1 dependent downstream signaling. As shown in Fig 3c, the adding of CP could attenuate phosphorylation of IGF-1R, AKT and ERK singling stimulated by IGF-1. Our data suggested that CP could down-regulate IGF-1R in H446 and H526 cells, which is probably through mAb-induced receptor endocytosis/degradation, and the endocytosed receptor could not significantly activate downstream signaling pathways compared with IGF-1. This nearly “silent” receptor down-regulation phenomenon might partially explain the anti-tumor effects of CP.

### Inhibition of ERK activation further enhances the therapeutic effect of CP to target SCLC cells

We then tried to combine CP with other drugs to increase its efficacy against SCLC. We noticed that there was some basal MEK/ERK activation in H446 and H526 cells after cell starvation and the pERK level was even slightly increased after CP treatment (Fig 3b). Given that the CP-induced ERK activation might underlie the mechanism of SCLC cell survival and drug resistance during CP therapy, we investigated the effects of combining CP with U0126, a specific MEK/ERK inhibitor to inhibit the downstream ERK activation, on H446 cell line. As shown in Fig 3d, cell viability kept decreasing with increasing concentration of U0126, suggesting a vital role of MEK/ERK signaling in SCLC. Furthermore, there was an additive effects of U0126 plus CP treatment compared with either U0126 only or CP only, implying a potential benefit of inhibiting ERK during CP therapy. And the western blot result showed the inhibitory effect of U0126 on MEK/ERK signaling. These findings are in keeping with Zinn. *et al*., who declared that low pretreatment p-ERK level may be indicative of sensitivity to IGF-1R small molecule inhibitor therapy in SCLC [29].

### Knockdown of ß-arrestin2 specifically inhibits MEK/ERK activation in CP-treated SCLC cells

We have shown that CP could induce IGF-1R degradation and ERK activation. We then try to investigate the mechanism of this IGF-1R auto-phosphorylation-independent ERK activation. As one of the main effects of CP were to induce IGF-1R endocytosis/degradation, we decided to focus on ß-arrestins (ß-arr), a key family of proteins involved in IGF-1R ubiquitination and endocytosis [30]. There are two ß-arrestin isoforms, ß-arr1 and 2, and they have been suggested to be functionally redundant [31,32]. We used siRNA to knockdown specific ß-arrestin (ß-arr1 KD or ß-arr2 KD) in H446 cells (Fig 3e), with scrambled siRNA as control. We first investigated their effects on IGF-1R degradation. KD of ß-arr1, but not ß-arr2, rescued the IGF-1-induced receptor degradation; however, KD of neither ß-arr1 nor ß-arr2 could inhibit CP-induced IGF-1R degradation (Fig 3f). We further studied the effects of ß-arrestins KD on ERK activation. Surprisingly, when we knocked down ß-arr1 in H446 cells, the pERK level was dramatically increased in both IGF-1- and CP-treated groups (Fig 3g), suggesting an inhibitory role of ß-arr1 during ERK activation. In contrast, in ß-arr2 KD cells the activation of ERK was lower in both IGF-1 and CP treated cells compared with control cells. Our data suggested that ß-arr2 was critical for ERK activation, and the combination of CP with ß-arr2 KD dramatically decreased the pERK below the detection level of Western blot, indicating potential therapeutic values to combine CP with ß-arr2 inhibitor to treat SCLC. Our results are also in accordance with published literature, where ß-arrestins were reviewed to scaffold the tyrosine kinase and could lead to the activation of ERK in G protein-coupled receptors (GPCRs)[33].

### Metformin inhibits SCLC through IGF-1R signaling pathway

**Fig 4:**
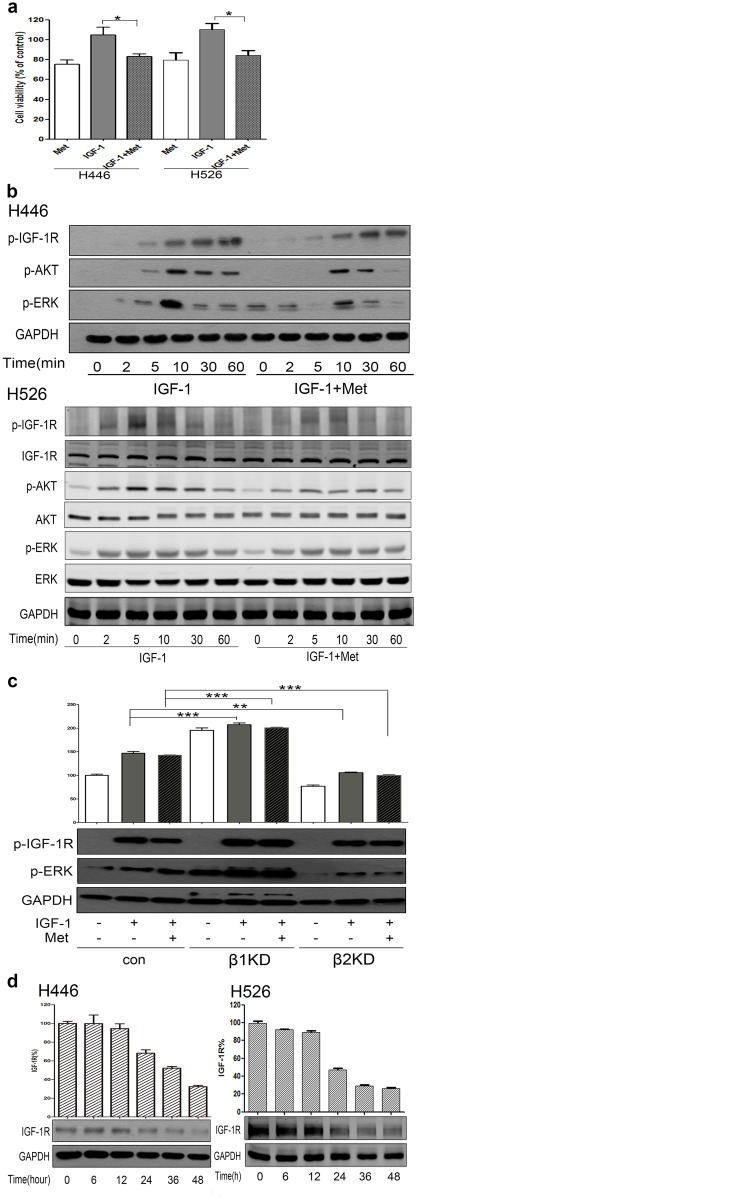
Metformin inhibits SCLC through IGF-1R signaling pathway. **(a)** H446 and H526 cells were treated 48h with metformin (3mM) alone, IGF-1 (6.5nM) alone, or IGF-1 plus metformin. Cell viability was tested via MTT. The number of viable cells following treatments is presented as percentage of untreated cells. **(b)** After 12h starvation, H446 and H526 cells were treated with or without 3mM metformin for 1h. Cells were then treated with 6.5nM IGF-1 for a series of time points. Cell lysates were analyzed via WB for p-IGF-1R, t-IGF-1R, p-AKT, t-IGF-1R, p-ERK, t-ERK and GAPDH. **(c)** ß-arr1 and ß-arr2 KD H446 cells were starved for 12h and treated with or without 3mM metformin for 1h. Cells were then stimulated with 6.5nM IGF-1 for 10min. Cell lysates were analyzed for p-IGF-1R, p-ERK and GAPDH through WB. **(d)** Cells were incubation with 3mM metformin for a series of time points, and the level of total IGF-1R was detected via WB. We did each experiment for three times, and data were represented as mean±SEM. Statistical analysis: *P<0.05, **P<0.01, ***P<0.001.

Next we asked if we could use CP together with another potential anti-cancer drug, metformin, to enhance its efficacy. We first tried to confirm the anti-tumor effects of metformin in SCLC by treating H446 and H526 cells with metformin alone or together with IGF-1(Fig 4a). Because IGF-1 stimulation attenuated the anti-proliferative effects of metformin, we speculated that metformin and CP might have additive anti-tumor effects. We also assessed the consequence of metformin on IGF-1R signaling pathway, and as shown in Fig 4b, the IGF-1-stimulated IGF-1R phosphorylation and the downstream PI3K/AKT and MEK/ERK activation were decreased after treating cells with metformin. Also, similar to previous results, KD of ß-arr1 promoted ERK activation while KD of ß-arr2 attenuated ERK activation, and these effects occurred with both IGF-1 only and IGF-1 plus metformin conditions in H446 cell line (Fig 4c). Furthermore, metformin also could down-regulate IGF-1R in a time-dependent manner in H446 cells (Fig 4d). Overall, although the precise mechanisms of metformin’s anti-cancer effects were elusive, our data suggested that it acts partially through inhibiting IGF-1R signaling pathway.

### Additive effects of CP with metformin to target SCLC

**Fig 5:**
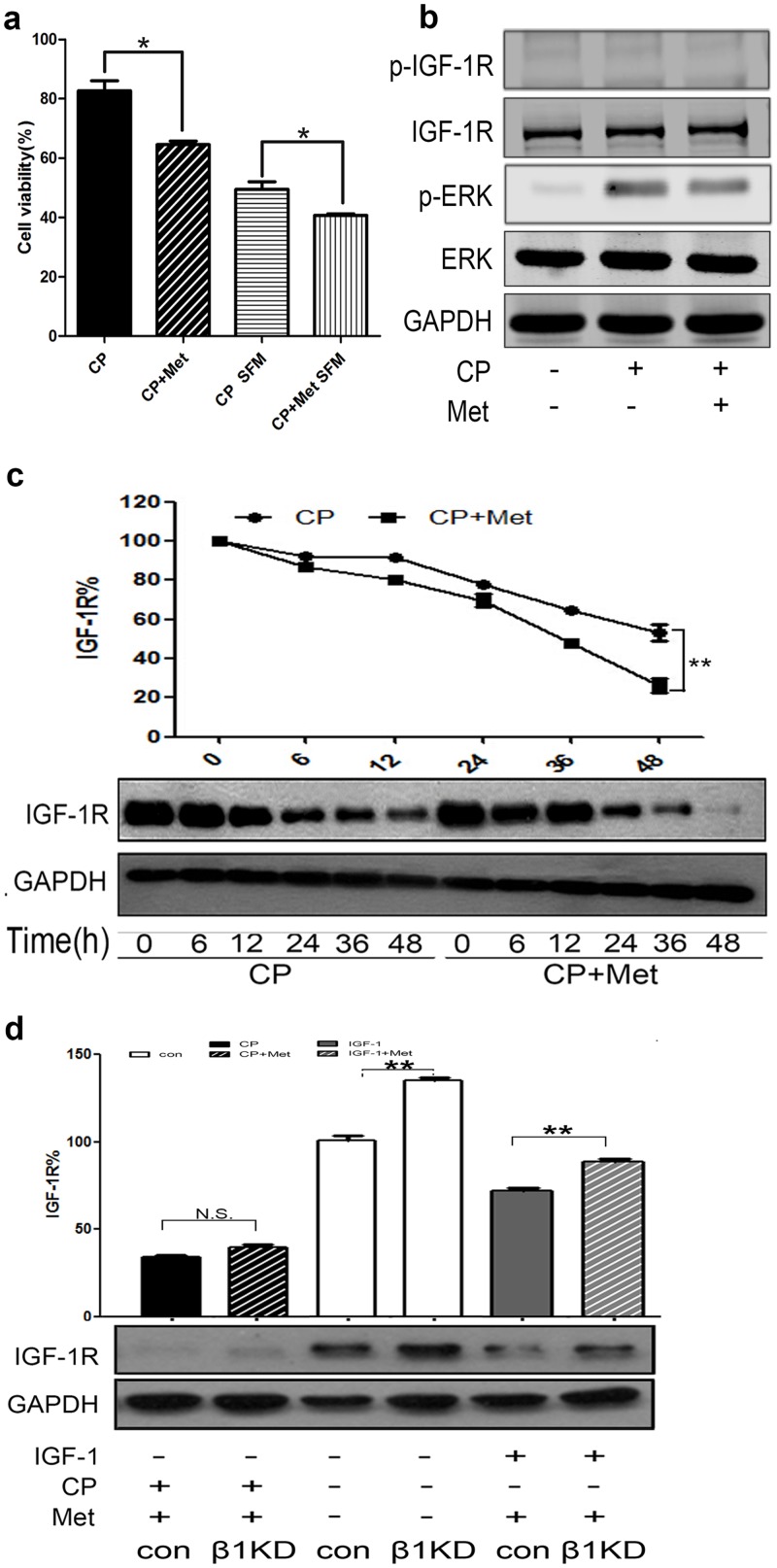
Metformin had additive anti-tumor effect with CP to target SCLC. **(a)** H446 cells were pre-treated with 3mM Metformin for 1h, and then 0.65nM CP was added with presence or absence of serum. MTT was conducted to test cell viability after 48h of incubation. The number of viable cells following treatment is presented as percentage of untreated cells. **(b)** After 12h starvation, cells were treated with or without 3mM of metformin for 1h, and then treated with 0.65nM CP for 10min. Cell lysates were analyzed for p-IGF-1R, t-IGF-1R, p-ERK, t-ERK and GAPDH via WB. **(c)** 3mM Metformin was added to cells for 1h, then 0.65nM CP was added for a series of time points. The expression level of IGF-1R and GAPDH were detected through WB. **(d)** ß-arr1 KD H446 cells were treated with 3mM metformin for 1h, and then incubated with 0.65nM CP for 24h. Levels of IGF-1R and GAPDH were detected via WB. We did each experiment for three times, and data were represented as mean±SEM. Statistical analysis: *P<0.05, **P<0.01, ***P<0.001.

Because the effects of metformin and CP were quite similar in terms of receptor down-regulation and IGF-1R signaling pathway inhibition, we then asked if there are any additive anti-tumor effects when combining metformin with CP. We tested H446 cell viability (MTT assay) after treatment with CP alone or CP plus metformin, and the experiment was performed both with and without serum. Similar to previous results, CP had more dramatic anti-proliferative effects in starved conditions compared with serum-containing conditions (Fig 5a). Intriguingly, metformin promoted CP’s effects against SCLC cells in both serum-containing (82.6% vs. 64.5%) and SFM (49.4% vs. 40.7%) conditions.

We then investigated the mechanism of this additive anti-cancer effect. Because CP alone stimulated pERK and we have shown some additive drug effects with MEK/ERK inhibitor, metformin might act similarly to help turn off ERK activation. However, although CP alone still increased pERK level, combining CP with metformin could not inhibit it (Fig 5b). Therefore, our results implied that the drug additive effect of metformin is different from that of MEK/ERK inhibitor. We then investigated whether metformin had some additive effects on receptor down-regulation. As shown in Fig 5c, time-dependent IGF-1R degradation was seen in both CP alone and CP plus metformin groups, and metformin could further enhance CP-induced receptor down-regulation. Also, similar to previous results, ß-arr1 KD could partially rescue the IGF-1 plus metformin induced IGF-1R down-regulation, but not for CP plus metformin (Fig 5d), while ß-arr2 KD could rescue neither (data not shown). Overall, our results suggested an additive therapeutic effect of CP with metformin to target SCLC. This combinational therapeutic effect might be through the induction of further IGF-1R down-regulation, and our data indicated that this process was regulated independent of ß-arrestins.

## Discussion

Antibody drugs are recently been widely used in cancer therapies. Although CP showed some encouraging results in phase II clinical trials to treat multiple cancers, the outcome of phase III clinical trial was disappointing. One of the main drawbacks of CP was lack of efficacy. Another recent clinical trial to test CP was prematurely terminated due to slow enrollment of patients. Therefore, currently there is a need to evaluate whether CP has enough therapeutic values to resume the clinical trial, and if so, how we could encourage the enrollment of patients. In the current study, we investigated the mechanism of CP and evaluated the potential of combination therapy to target SCLC.

We examined across different SCLC patient tissues and consistent with precious results, we found patients with higher IGF-1R expression tumors had a poorer survival rate (Fig 1b), suggesting a potential therapeutic value of CP to target SCLC. Similar to SCLC patient tissues, H446 and H526 cells have relatively high expression of IGF-1R (Fig 2a), and CP treatment could inhibit the proliferation of H446 and H526 cells in both serum-added and serum-free conditions (Fig 2b). The anti-proliferative effect in serum free conditions was surprising because the IGF-1R was generally unphosphorylated and inactive without serum and the main effect of CP was thought to compete with IGF-1 to bind/inhibit IGF-1R. Our result implied that beside as an IGF-1 competitor, CP could also inhibit SCLC through a mechanism that is independent of IGF-1R auto-phosphorylation. We found CP could induce IGF-1R endocytosis/down-regulation, which was more potent than that induced by IGF-1, and the CP-induced endocytosed IGF-1R kept unphosphorylated and inactive (Fig 3b). Therefore, one possible explanation could be the endocytosed unphosphorylated IGF-1R might trigger other signaling pathways that promote the anti-proliferative/apoptosis effects. However, this hypothesis should be validated in future studies.

Although CP could not activate PI3K/AKT that is downstream of the IGF-1R signaling pathway, we demonstrated that CP could promote ERK activation, which was mediated via ß-arrestin2 while inhibited by ß-arrestin1. The actual level of CP-induced ERK activation might be balanced by the amount of ß-arrestin1 and 2 in the cells. Our finding is in keeping with previous studies that declared the role of ß-arrestin2 in activating ERK in GPCRs [32–34]. In contrast, this finding is disagree with Girnita *et cl*, who found CP could activate ß-arrestin1 dependent ERK signaling in Ewing's sarcoma cell lines [35]; however, this discrepancy might be due to the different histologic origin of tumors. Similar results were seen in some studies in the field of GPCRs while the exact role of ß-arrestin isoforms varies due to different receptors and tumors [36]. Therefore, due to the complex function of ß-arrestins in different cancers and signaling pathways, the role of different isoforms should be carefully studied before using them as drug targets.

There are at least two side effects of CP-based therapy: 1) CP could stimulate ERK activation and thus might promote cancer cell survival; 2) Hyperglycemia is one of the most highly occurred adverse events in clinical trials, which might benefit tumor cell growth. To offset the side effects and increase the efficacy of CP-based therapy, combinational treatment might be necessary. We tested co-treatment of CP with either the MEK/ERK inhibitor or metformin, and both of them showed additive effects to target SCLC cells. MEK/ERK signaling pathway plays critical roles in the regulation of many cellular processes, and combining MEK/ERK inhibitors with other drugs are frequently performed during cancer therapy. The MEK/ERK inhibitor AZD6244 has been shown to have more benefits to the biliary cancer patients when treated after gemcitabine rather than concurrently [37]. Therefore, even though we saw beneficial effects of combining CP with MEK/ERK inhibitor, the actual drug scheme might be important and further clinical trials are necessary to resolve this question.

In our studies, metformin also showed significant therapeutic values when combining with CP to treat SCLC. Multiple studies have been shown that metformin has anti-tumor properties. Despite of its metabolic effects, metformin was reported to affect IGF-1R and the downstream signaling pathways [17,38], although the precise mechanism is not yet well illustrated. Metformin has been demonstrated to have adjuvant effects with some chemotherapeutic drugs [39]. In our study, we tested metformin’s anti-tumor effects and we are the first group who attempts to combine metformin with an IGF-1R mAb and confirm their additive effects to target SCLC cells. Moreover, our data suggested that it is the IGF-1R signaling inhibition/receptor down-regulation underlying the anti-tumor effects of metformin. Also, since metformin could lower blood glucose level, which could offset one of the main side effects of CP, the actual benefits of combining CP with metformin might be higher in clinical trials. Therefore, the future research and clinical studies are both needed to fully illustrate the therapeutic values of CP-based combination therapy to target SCLC.

Altogether, the current study evaluated the effects of CP to target SCLC cells. Our data suggested that CP inhibits SCLC cell proliferation through promoting IGF-1R down-regulation without significant downstream signaling activations. We also illustrated that after combining CP with either MEK/ERK inhibitor or metformin, it could significantly increase the efficacy of CP to inhibit SCLC. Our studies help further demonstration of the therapeutic value of CP to target SCLC and encourage the use of combinational therapy for antibody drugs. Overall, our results could help to direct the future design of clinical trial to test anti-IGF-1R mAbs in SCLC and other cancers.
